# Phytochemicals Analysis, *In Vitro* Antibacterial Activities of Extracts, and Molecular Docking Studies of the Isolated Compounds from *Melhania zavattarii* Cufod Leaves

**DOI:** 10.1155/2023/8820543

**Published:** 2023-06-01

**Authors:** Teshome Gonfa, Ayalew Temesgen, Olyad Erba, Ephriem T. Mengesha, Muthusaravanan Sivasubramanian

**Affiliations:** ^1^Department of Chemistry, College of Natural and Computational Sciences, Haramaya University, P.O. Box 138, Dire Dawa, Ethiopia; ^2^School of Biological Sciences and Biotechnology, College of Natural and Computational Sciences, Haramaya University, P.O. Box 138, Dire Dawa, Ethiopia

## Abstract

*Melhania zavattarii* Cufod is an endemic plant species to Ethiopia and is used to treat ailments related to kidney infection. The phytochemical composition and biological activity of *M. zavattarii* have been not reported yet. Therefore, the present work aimed to investigate phytochemical constituents and evaluate the antibacterial activity of different solvents' leaf extracts and analyze the molecular binding capacity of isolated compounds from the chloroform leaf extract of *M. zavattarii*. Accordingly, preliminary phytochemical screening was tested by using standard procedures and the result indicated that phytosterols and terpenoids as major and others like alkaloids, saponins, flavonoids, tannins, phlobatannin, and coumarins were detected as minor in extracts. Antibacterial activity of the extracts was evaluated using the disk diffusion agar method, and the activities revealed that chloroform extract showed the highest inhibition zones, 12.08 ± 0.38, 14.00 ± 0.50, and 15.58 ± 0.63 mm against *Escherichia coli* at 50, 75, and 125 mg/mL concentrations, respectively, compared to that of *n*-hexane and methanol extracts at respective concentrations. Methanol extract showed the highest zone of inhibition 16.42 + 0.52 against *Staphylococcus aureus* at 125 mg/mL concentration compared to that of *n*-hexane and chloroform extracts. Two compounds, namely, *β*-amyrin palmitate (**1**) and lutein (**2**) were isolated and identified for the first time from the chloroform leaf extract of *M. zavattarii*, and structural elucidations of these compounds were accomplished by using spectroscopic methods (IR, UV, and NMR). For the molecular docking study, 1G2A, which is a protein of *E. coli* and chloramphenicol standard target, was selected. Binding energies of −9.09, −7.05, and −6.87 kcal/mol were calculated for *β*-amyrin palmitate, lutein, and chloramphenicol, respectively. The drug-likeness property result indicated that both *β*-amyrin palmitate and lutein violated two rules of Lipinski's rule of five with molecular weight (g/mol) > 500 and LogP > 4.15. In the near future, further phytochemical investigation and biological activity evaluation should be conducted on this plant.

## 1. Introduction

Over the past decade, traditional medicinal plants have been closely linked and played important roles in the treatment of human illnesses in the world [1]. According to World Health Organization (WHO) estimation, more than 80% of the total world population in developing countries use mostly plant parts as traditional medicine for their primary health care [2]. Still now, medicinal plants have been receiving significant attention [3] since they possess antimicrobial, antioxidant, antimalarial, anti-inflammatory, antiemetic, antidiabetic, antifertility, antiasthmatic, antistress, and anticancer activities [4]. It is well-known that the medicinal values of these plants are due to some bioactive compounds that produce a definite physiological action on the human body since plants synthesize extremely diverse chemical compounds which have great potential for the development of new pharmaceuticals [5].

Even though nearly 7000 higher plant species and 1000 medicinally valued plants are expected to be found in the flora of Ethiopia [6, 7], the scientific research works done are more on ethnobotanical studies which remained in listing and surveying of many plant species. The research works done on the phytochemical constituents and biological activities of Ethiopian medicinal plants were minimal and limited to a few plant species. It is therefore very apparent to conduct an extensive scientific investigation on the phytochemical and antimicrobial activity of untouched medicinal plants of Ethiopia to support their traditional therapeutic uses, which may also lead to a search for some new bioactive drugs. There is a need for the screening of more effective, affordable, and readily available antimicrobial substances from local medicinal plants or herbs as pathogenic bacteria are developing resistance to common antibiotics [8].

*M. zavattarii* (locally known as muka bira in Afan Oromo) is a shrub plant, which belongs to the family *Sterculiaceae*. It is an endemic plant species to Ethiopia, which is found mainly in the eastern and southern parts of the country. *M. zavattarii* exhibits simple, hairy, margin serrate leaf, and axillary and solitary flowers. The leaf of the plant is used to treat ailments related to kidney infection in the eastern part of Ethiopia [9]. To the best of our knowledge, no research work has been reported so far on the chemical constituents and antimicrobial activities of this endemic plant species. Hence, the present study is intended to screen major phytochemical classes, investigate antibacterial activities of the crude extracts, and isolate compounds from the chloroform leaf extract of *M. zavattarii*. Furthermore, the structures of the isolated compounds were identified based on spectroscopic analysis using UV-VIS, FT-IR, and NMR and comparison with values reported in the literature. Finally, the molecular binding capacity of the compounds was studied by docking against IG2A protein model.

## 2. Materials and Methods

### 2.1. Plant Materials

**Figure 1:**
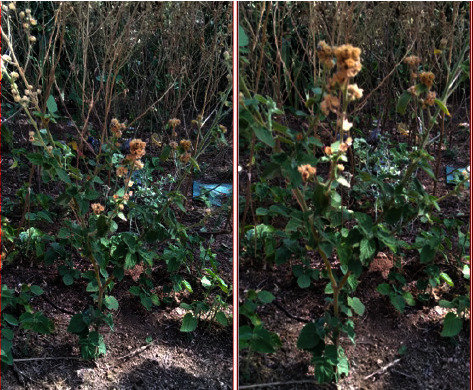
Muka bira (*Melhania zavattarii* Cufod), photo was taken by researchers in December 2021.

The leaf of *M. zavattarii* Cufod (Figure 1) was collected from Harla town and its surrounding areas in December 2021. Harla is found in Dire Dawa administrative city, 515 km far from the capital Addis Ababa, Ethiopia. Identification of the plant was previously reported with voucher number AHU127 at the herbarium of Haramaya University, Ethiopia [9]. The collected leaf was washed and dried under shade at room temperature and pulverized into a fine powder using an electric grinder (Shanghai Jinkle, Scientific Instrument Co., Ltd., China).

### 2.2. Chemicals and Apparatus

Dichloromethane (DCM) RS, *n*-hexane RS (for HPLC-isocratic), and methanol (MeOH, 99%) were purchased from Carlo Erba reagents (France), ethyl acetate (EtOAc, 99.8% for HPLC and UV spectroscopy), and chloroform (99.8% AR) were obtained from Loba Chemie Pvt., Ltd. (India), while Mueller–Hinton agar (MHA) and dimethyl sulfoxide (DMSO) were obtained from Micro Express, India. Thin layer chromatography (TLC) aluminium sheet (20 × 20 cm) and silica gel (100–200 mesh size for column chromatography) were purchased from Loba Chemie Pvt., Ltd.). The rotary evaporator (Rotary Vacuum, Jainsons, India) was also employed in this study.

### 2.3. Instrumentation

Stuart SMP3 melting point apparatus determined the melting point. UV-Vis spectra were recorded on Genesys 2PC UV-Vis scanning spectrometer (200–800 nm) in chloroform. The IR spectral data were recorded with KBr pellets on Perkin Elmer Bx infrared spectrometer in the range of 400–4000 cm^−1^. Optical rotations were measured on ADP 220 polarimeter at 25° in CHCl_3_. All the NMR analyses were performed on Bruker Advance 400 spectrometer (in CDCl_3_) at 400.13 MHz for the ^1^H and 100.6 MHz for the ^13^C and dept-135 spectra. The residual proton signal of the solvent was used as a reference, and the chemical shift was expressed by *δ* (ppm).

### 2.4. Preparation of Crude Extracts and their Percentage Yields

To investigate the effect of solvents on the extraction yields, powder leaf of *M. zavattarii* (80 g) was extracted separately with *n*-hexane, chloroform, ethyl acetate, and methanol (600 mL, each) by shaking on an orbital shaker at room temperature for 48 hrs. The extracts were then filtered using Whatman's No.1 filter paper (Whatman International Ltd., England), and the filtrates were concentrated using a rotary evaporator under reduced temperature and pressure. The corresponding percentage extract yield (%) was calculated using the following formula [10]:

(1) $$\begin{array}{c} Extract\;yield\;\left(\%\right)=\frac{Weight\;of\;crude\;extract}{Weight\;of\;powdered\;sample}\times 100\text{.} \end{array}$$

### 2.5. Phytochemical Screening Test

The *n*-hexane, chloroform, ethyl acetate, and methanol leaf extracts were subjected to various phytochemical screening tests following the standard procedures [11, 12] with some modifications to detect the presence or absence of major phytochemical classes, namely, flavonoids, alkaloids, phytosterols, tannins, terpenoids, saponins, free anthraquinone phlobatannin, quinines, and coumarin. The screening analysis was monitored through the observation of the color change and a precipitate formation after the addition of specific reagents.

### 2.6. Antibacterial Activity Test

Three bacterial species namely, *Escherichia coli, Staphylococcus aureus* and *Pseudomonas aeruginosa* were obtained from Ethiopian Biodiversity Institute based on their availability and relatedness to ethnomedicinal use of the plants. Antimicrobial assay of the extracts was conducted following the standard Mueller–Hinton agar disc diffusion method [8, 13, 14] for the obtained bacterial pathogen. A stock solution of each crude extract (50, 75, and 125 mg/ml) was prepared in DMSO and paper discs of 6 mm diameter were impregnated with 0.01 ml of the extracts. Commercial chloramphenicol antibiotics discs (5 *μ*g) were used as positive control, and the pure solvent (DMSO) impregnated discs were used as negative control. The diameters of the zone of inhibition around each disc was measured to the nearest millimeter along two axes (i.e., 90° to each other) by using a transparent ruler, and the means of the two readings were recorded.

### 2.7. Fractionation and Isolation of Compounds

The powder (300 g) of *M. zavattarii* leaf was defatted with *n*-hexane (2.5 L, 3x) for 48 hrs on an orbital shaker. The remaining residue, after *n*-hexane extraction, was then extracted successively with chloroform and methanol solvents for 48 hrs. The filtered and concentrated solvents by using rotary evaporator under reduced pressure afforded 1.92 g, 8.25 g, and 9.48 g of crude extracts of *n*-hexane, chloroform, and methanol extracts, respectively. Then after, all extracts were subjected to the TLC analysis using different ratios of *n*-hexane/EtOAc and DCM/EtOAc solvent system and visualizing techniques UV-lamp (254 and 365 nm) and iodine vapor. Based on the phytochemical screening and TLC profile results, the chloroform extract was subjected to silica gel column chromatography (CC) as follows.

Part of the chloroform extract (5 g) was dissolved in chloroform, adsorbed on silica gel (35 g), and concentrated and applied on CC silica gel. The elution was carried out using the eluents *n*-hexane/EtOAc and DMC/EtOAc with different polarity ratios, which resulted in the collection of 90 fractions (15–25 mL). Collected fractions were subjected to TLC examination using different ratios of hexane/EtOAc and DMC/EtOAc solvent systems and UV-lamp and iodine vapor as detecting methods. Fractions with similar *R*_*f*_ values and TLC profiles were combined and resubjected to the TLC analysis. Among them, fraction 9–23 (eluted with 85 : 15 of *n*-hexane/EtOAc) and fraction 81–88 (eluted with 70 : 30 of DCM/EtOAc) showed a relatively better purity on TLC and fractionated further over silica gel CC as follows.

Fraction 9–23 (1.2 g) was dissolved in chloroform, adsorbed in silica gel (7 g), and concentrated and purified over silica gel CC which led to the collection of 27 subfractions with the eluent *n*-hexane/EtOAc of different ratios. After TLC examination, subfraction 21–23 (eluted with 9 : 1 of *n*-hexane/EtOAc) presented a single spot with *R*_*f*_ value 0.5 using 19 : 1 *n*-hexane/EtOAc (19 : 1) as the solvent system and UV-lamp (254 and 365 nm) and iodine vapor which afforded yellow amorphous compound **1** (26 mg). Similarly, fraction 81–88(0.10 g) was further chromatographed over silica gel CC with different ratios of DCM/EtOAc resulted in the collection of 33 subfractions. Of which, subfraction 25–27 (eluted with 80 : 20 of DCM/EtOAc) exhibited a single spot (*R*_*f*_ value of 0.6) on TLC developed with DCM/EtOAc (4 : 1) and visualized under UV-lamp and iodine vapor which furnished a brick-red crystalline solid compound **2** (23 mg). The structures of the isolated compounds **1** and **2** were established based on the spectroscopic analysis using UV-Vis, IR, ^13^C, and ^1^HNMR and comparison with values in the literature.

### 2.8. Molecular Docking Analysis

The aim of the docking study was to analyze the binding interaction of the isolated compounds with one of the chloramphenicol's targets, peptide deformylase (PDB ID: 1G2A) [15]. AutoDock 4.2 program [16] was used for the docking study of the compounds **1** and **2** with the peptide deformylase enzyme (PDF) (PDB ID: 1G2A). Water molecules and ligands were deleted from the receptor followed by the addition of polar hydrogens and Kollman partial charges using Autodock tools. A grid box with spacing 0.375 Å, size of 60 × 60 × 60 along the *X*, *Y*, and *Z*-axes and Lamarckian genetic algorithm was used. LigPlot [17] was used to elucidate the two-dimensional projection of the ligand-target interactions. Ground state geometry of the ligands, compounds **1** and **2** were optimized using Gaussian 09 program package [18]. Swiss ADME, PreADMET, and ProTox-II property explorer software, also predicted the drug-likeness, ADME and toxicity characteristics of the isolated compounds **1** and **2**.

## 3. Results and Discussion

### 3.1. Percentage Extraction Yield

In the present study, the percentage extraction yield of *n*-hexane, chloroform, ethyl acetate, and methanol solvents extract of *M. zavattarii* leaf was determined. Methanol extract recorded the highest percentage extract yield (8.42%) followed by chloroform extract (3.24%) and ethyl acetate extract (1.59%), whereas the least extract yield was obtained from *n*-hexane (0.95%). The result indicated that the extraction efficiency favors the highly polar solvents.

### 3.2. Phytochemical Composition of Leaf Extracts

The presence or absence of flavonoids, alkaloids, tannins, terpenoids, saponins, free anthraquinone, phytosterols, quinines, phlobatannin, and coumarin was screened for leaf extracts of *M. zavattarii.* The extracts showed the presence and absence of certain phytochemical classes, as summarized in Table 1.

As shown in Table 1, flavonoids, terpenoids, saponins, phytosterols, and coumarin were detected in both chloroform and methanol extracts, whereas alkaloids, free anthraquinones, and quinines were not detected in the extracts. Only alkaloid and phytosterol, and terpenoid and phytosterol were detected in ethyl acetate and *n*-hexane extracts, respectively. The phytosterols were moderately detected in all extracts using the Lieberman Bouchard test, whereas the free anthraquinone and quinine were screened negatively in all extracts using the Borntrager's and ammonium hydroxide tests. The chloroform and methanol extracts, respectively, confirmed a moderate and strong presence of flavonoids and saponins. Terpenoids were strongly detected both in the *n*-hexane and in chloroform extracts, whereas tannins were strongly screened only in the methanol extract.

### 3.3. Antibacterial Activity of Leaf Extracts

The disc diffusion method was used to determine antibacterial activity of *n*-hexane, chloroform, and methanol crude extracts against *E. coli*, *S. aureus*, and *P. aeruginosa*, and the results are presented in Table 2.

As can be seen from Table 2, *P. aeruginosa* was found resistant to all types of extracts at all concentrations. Compared to the standard chloramphenicol types of extracts at all concentrations, *P*. *aeruginosa* showed lower antibacterial activity. Chloroform extract exhibited higher mean diameter zone inhibition (12.08, 14.00, and 15.58 mm) than hexane (7.67, 11.08, and 14.75 mm) and methanol (7.00, 8.75 and 11.00 mm) against *E. coli* at concentration of 50, 75, and 125 mg/mL, respectively. On the contrary, *n*-hexane extract exerted the highest activity (10.33, 12.92, and 14.08 mm of the mean zone of inhibition) against *S. aureus* than chloroform extract with diameter zone inhibition (7.00, 7.17, and 10.60 mm) and methanol extract (7.25, 11.08, and 16.42 mm), respectively, against *S. aureus*. The antibacterial activity of the leaf extracts of *M. zavatarii* in the present study might be attributed to the phytochemical constituents of the extracts (Table 1). Only terpenoids and phytosterols were detected in *n*-hexane extracts, but it showed comparable antibacterial activity to chloroform and methanol extracts that had more phytochemical constituents. The differences in the antimicrobial activities of the various extracts might be due to various solvents having different solubility for different phytoconstituents [19]. The quantity of terpenoids might be the reason of this finding. In line to this, the composition and quantity of alkaloids, saponins, tannins, and terpenoids were associated with antibacterial activities against *Salmonella typhi*, *Shigella boydii*, *S. aureus*, and *Enterococcus faecalis* [8].

### 3.4. Structural Elucidation of Isolated Compounds **1** and **2**

Two compounds (compounds **1** and **2**) were isolated for the first time from the chloroform leaf extract of *M. zavattarii.* Compound **1**: it was obtained as a yellow amorphous (26 mg) with melting point of 69-70°C which is comparable with the literature reported melting point 72-73°C [20] and has an *R*_*f*_ value of 0.5 (*n*-hexane/EtOAc; 19 : 1). The compound was not readily visible on TLC plates under UV_254_/_365_ nm lamp but visualized and detected as yellow color following exposure to iodine vapor. The optical rotation ([*α*]_D_^23^, *c* = 0.85 in CHCl_3_ as a solvent) was found to be +67.5° indicating that the compound is optically active. The UV-Vis spectrum, in CHCl_3_ showed two absorption maxima (*λ*_max_) at 240 and 270 nm suggestive of the presence of a carbonyl and double bond groups. The IR spectrum displayed strong absorption bands at 2918 and 2848 cm^−1^ and indication of the typical C-H bond stretching of alkyl groups. Besides, absorption bands at 1735 and 1172 cm^−1^ were observed in the IR spectrum which corresponded to the C=O and C-O stretching of the normal aliphatic esters, respectively.

The ^1^H NMR spectrum showed two multiplet signals at *δ*_H_ 5.19 (1H, H-12) and 4.50–4.54 (1H, H-3) indicating the presence of an olefinic methine proton and oxygenated methine proton of a *β*-hydroxyl esterified pentacyclic triterpene, olean-12-ene. Besides, a triplet of doublet signal at *δ*_H_ 2.31(2H, H-3′, td, *J* = 7.8, 3) and an overlapped multiplet signal at *δ*_H_ 1.27 (24H, H-4′ to -15′) which integrated to 24 protons suggested the presence of *α*-methylene and fatty acid straight chain, respectively. The ^1^H spectrum also displayed five singlet signals at *δ*_H_ 0.85–1.15 which attributed to eight methyl groups (H-23 to H-30) and a multiplet signal of a methine proton at *δ*_H_ 0.89 (1H, H-5) which was another evidence to the general skeleton of compound **1** to be a pentacyclic triterpene.

The ^13^C and DEPT-135 spectra (Table 3) displayed a down shifted signal at *δ*_C_ 173.7 (C-1′) attributed to an ester carbonyl carbon of esterified palmitic acid along with its aliphatic carbon signals appeared *δ*_C_ 34.7 (C-2′), 25.1 (C-3′), 29.2–29.7 (C-4′ to C-13′), 31.9 (C-14′), 23.5 (C-15′), and 14.1 (C-16′). The carbon signals observed at *δ*_C_ 121.7 (C-12) and 145.2 (C-13) belong to the olefinic methine (corresponded to the proton signal at *δ*_H_ 5.19) and quaternary carbons of the olean-12-en pentacyclic triterpene core. In addition, the existence of an oxygenated methine carbon signal at *δ*_C_ 79.0 (C-3), which corresponded to a multiplet oxymethine proton signal at *δ*_H_ 4.55−4.49 (H-3), affirmed that the palmitic acid moiety was esterified to the olean-12-en pentacyclic triterpene core at C-3.

**Figure 2:**
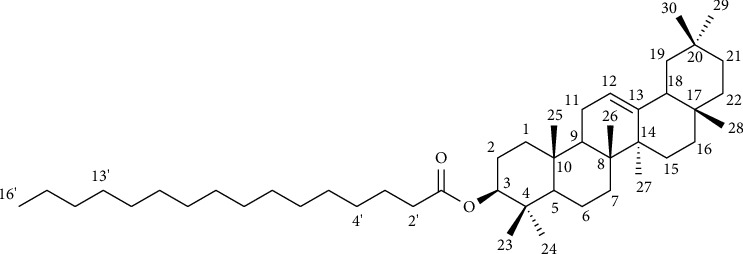
Structures of compound **1** (*β*-amyrin palmitate).

Furthermore, ten methylene carbon signals at *δ*_C_ 38.3 (C-1), 22.7 (C-2), 18.3 (C-6), 32.6 (C-7), 23.6 (C-11), 26.9 (C-15), 26.1 (C-16), 47.6 (C-19), 34.9 (C-21), and 37.2 (C-22) and five quaternary sp^3^ hybridized carbon signals at *δ*_C_ 37.75 (C-4), 39.8 (C-8), 36.8 (C-10), 32.5 (C-17), and 31.1 (C-20) were identified in the ^13^C and DEPT-135 spectra which fitted to the olean-12-en pentacyclic triterpene. All the NMR assignments of compound **1** and values in the literature for a similar structure are presented in Table 3. Accordingly, the obtained NMR data of compound **1** were found closely matched with the values reported in the literature for *β*-amyrin palmitate [21, 22]. Based on this, the structure of compound **1** was established as *β*-amyrin palmitate (Figure 2).

Previously, *β*-amyrin palmitate was isolated from different plant species [22, 23]. It exhibits a wide range of pharmacological and biological activities, cytotoxicity against breast cancer cells [20], antidiabetes [23], antidepressants [24], anti-inflammatory [21], and antidyslipidemic [22].

Compound **2** was afforded as a brick red solid (23 g) with melting point 189–192°C which is comparable with the literature reported melting point 190°C [25, 26], *R*_*f*_ value 0.6 (4 : 1 DCM/EtOAc), and optical rotation ([*α*]_D_^23^, *c* = 0.25 in CHCl_3_) +37.6°. The resulting UV spectrum showed four absorption peaks at 225, 262, 410, and 655 nm indicating that compound **2** exhibited a conjugated structure. The IR spectrum provided an intense and broad absorption bands at 3351 and 2923−2856 cm^−1^ which were due to the vibrational stretching of hydroxyls (OHs) and aliphatic -CH groups. The bands at 1440 and 1360 cm^−1^, in the IR spectrum, were due to the C-H bending vibrations and the medium 1022 and 962 cm^−1^ were due to the C-H out of plane bending of *trans*-conjugated alkenes.

The acquired ^1^H spectrum showed nine singlet signals attributed to ten methyl groups; four attached to sp^3^quaternary carbons and appeared at *δ*_C_ 1.07 (6H, H-16, and 17), 0.84 (3H and H-16′), and 1.00 (3H, H-17′); and six attached to olefinic carbons and occurred at *δ*_H_ 1.75 (3H and H-18), 1.63 (3H and H-18′), 1.92 (3H and H-19′), and 1.97 (9H, H-19, H-20, and H-20′). Besides, the proton spectrum displayed two singlet signals (integrated to one proton each) at *δ*_H_ 4.02 (1H and H-3) and 4.27 (1H and H-3′) which corresponded to two terminal oxymethinic protons. Furthermore, the presence of a multiplet signal at *δ*_H_ 6.60–6.69 (4H, H-11, H-11′, H-15, and H-15′), two doublet signals at *δ*_H_ 6.38 (2H, H-12, and H-12′, d, *J* = 14.5), and doublet signal at *δ*_H_ 6.27 (2H, H-14, and H-14′, d, *J* = 8.3), two multiplet signals at *δ*_H_ 6.18 (3H, H-8′, H-10, and H-10′) and 6.14−6.11 (2H, H-7, and H-8), a singlet signal at *δ*_H_ 5.56 (1H and H-4′) and a doublet of doublet signal at *δ*_H_ 5.44 (1H, dd, H-7′, *J* = 15.4, 9.9) suggesting to the existence of a conjugated carotenoid-type of structure.

The ^13^C and DEPT-135 spectra provided thirty-five well-resolved resonances which ascribed to forty carbons, ten methyl carbons appeared at *δ*_C_ 30.3 (C-17), 29.5 (C-17′), 28.7 (C-16), 24.3 (C-16′), 22.9 (C-18′), 21.6 (C-18), 13.1 (C-19′), 12.8 (C-20 and C-20′), and 12.8 (C-19); three methylene carbons at *δ*_C_ 48.4 (C-2), 42.5 (C-4), and 44.7 (C-2′); one methine carbon at *δ*_C_ 55.0 (C-6′); two hydroxylated carbons at *δ*_C_ 65.1 (C-3) and 65.9 (C-3′); nine quaternary carbons at *δ*_C_ 37.1 (C-1), 34.1 (C-1′), 126.2 (C-5), 138.0 (C-6 and C-5′), 135.7 (C-9), 136.5 (C-13), 135.1 (C-9′), and 136.4 (C-13′); and fifteen conjugated olefinic carbons at *δ*_C_ 124.5 (C-4′), 125.6 (C-7), 138.5 (C-8 and C-8′), 131.3 (C-10), 124.9 (C-11), 137.8 (C-12), 132.6 (C-14 and C-14′), 130.1 (C-15 and C-15′), 137.6 (C-12′), 124.8 (C-11′), 130.8 (C-10′), and 128.7 (C-7′) which highlighted the presence of xanthophyll carotenoid type of structure. The overall obtained spectral data of compound **2** was found in a good agreement with the values in the literature for the lutein compound [27, 28] (Table 4).

**Figure 3:**
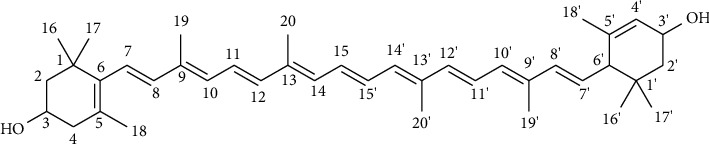
Structures of isolated compound **2**.

Accordingly, the structure of compound **2** was as lutein (Figure 3).

Lutein (tetraterpenoid class of natural carotenoid) was reported to possess anti-inflammatory and antioxidant [29], cytotoxicity [27] antitumorigenic, antiangiogenic, photoprotective, hepatoprotective, neuroprotective, and acrolein-induced ototoxicity preventing properties [30, 31]. Even though *β*-amyrin palmitate and Lutein have been isolated from different plant parts, this is the first time a report of isolation of the two compounds from *Melhania zavattarii* is being made. The reviewed evidence indicated the plant might exhibit a broad range of pharmacological activities, such as antidepressants, antidiabetes, antioxidants, anti-inflammatory, and cytotoxicity.

### 3.5. Molecular Docking Study

**Figure 4:**
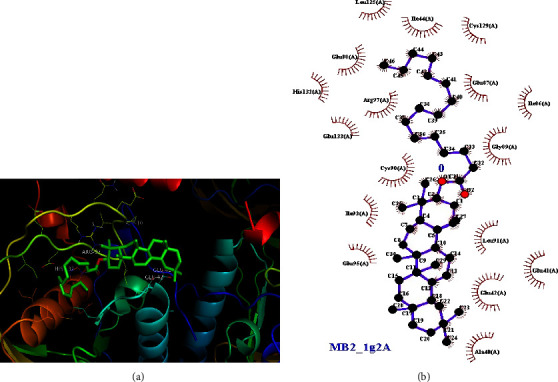
(a) Docking interaction of compound **1** with 1G2A protein of *E. coli*. (b) 2-D projection of the interaction plotted using LigPlot. The calculated binding interaction of compound **1** with 1G2A is −9.09 kCal/mol.

**Figure 5:**
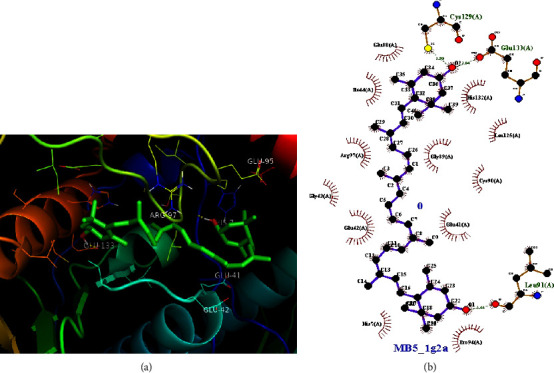
(a) Docking interaction of compound **2** with 1G2A protein of *E. coli*. (b) 2-D projection of the interaction plotted using LigPlot. The calculated binding interaction of compounds **2** with 1G2A is −7.05 kCal/mol.

**Figure 6:**
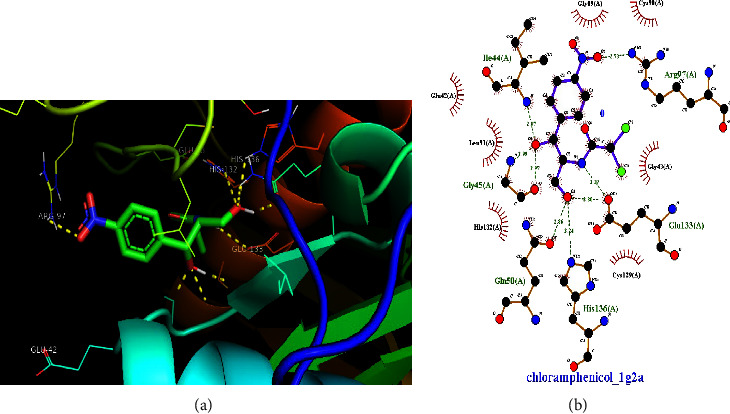
(a) Docking interaction of chloramphenicol with 1G2A protein of *E. coli*. (b) 2-D projection of the interaction plotted using LigPlot. The calculated binding interaction of chloramphenicol with 1G2A is −6.87 kCal/mol.

As presented in the previous section (Table 2), the crude chloroform extract of *M. zavattari* showed the best activity against *E. coli,* which is close to half the activity of the standard chloramphenicol (inhibition zone 14 mm vs. 26.78 mm). In the present study, the docking study aimed to find evidence whether the isolated compounds contribute to the observed antibacterial activity of the crude extracts against *E. coli*, or not. Figures 4–6 show the docking interactions of the isolated compounds and the standard with 1G2A protein target. Binding energies of −9.09, −7.05, and −6.87 kCal/mol (Table 5) are calculated for compounds **1**, **2**, and chloramphenicol, respectively. The strong binding interaction of the two compounds with target protein could suggest possible activity of these isolated compounds against *E. coli*. It is interesting to note that the standard shows more antibacterial activity despite smaller binding energy relative to the two extracts. This might be due to the presence of specific interactions, which are critical for the antibacterial activity. Further study is required for elucidated specific interaction present between standard and target so that the observed difference in antibacterial activity will be accounted. From the two-dimensional projections of the interactions, it is visible that the standard shows far greater number of specific hydrogen bond interaction with the protein residues of the target than the two compounds. A similar situation is encountered in the work of [32] where good antitubercular activity of ligands are observed as a result of specific interactions with important protein residues despite smaller binding energies.

### 3.6. Pharmacokinetic Properties Study of Isolated Compounds **1** and MB **2**

The results of the pharmacokinetic properties studies are presented in Tables 6–8 below. The drug-likeness property results (Table 6) indicated that both the isolated compounds **1** and **2** violated two rules of Lipinski's rule of five with molecular weight (g/mol) > 500 and LogP > 4.15. The ADME prediction result (Table 7) revealed that both the isolated compounds, unlike to chloramphenicol, showed a low degree of gastrointestinal absorption and interacted with the P-glycoprotein substrate, whereas similar to the chloramphenicol, both compounds were found as nonpermeable to the blood brain barrier and noninhibitors of all the predicted cytochrome-P enzymes. These two isolated compounds were found within the toxicity classes of 4 and 2 with LD_50_ (mg/kg) values of 339 and 10, respectively; and both exhibited an immune toxicity property (Table 8).

## 4. Conclusion

The results of the present investigation showed that the highest yield of leaf extracts was achieved by methanol followed by chloroform solvent used for maceration extraction from *Melhania zavattarii* leaf, which could be preferred for further investigation on additional bioactive phytochemical constituents. Furthermore, methanol and chloroform extracts were enriched with phytosterols, terpenoids, flavonoids, saponins, tannins, and coumarin as active phytochemical constituents. It seems that the presence of diverse phytochemicals attributed to the antibacterial activity of methanol and chloroform extracts against *E. coli* and *S aureus*. However, *n*-hexane with only terpenoids and phytosterols major class did show comparable antibacterial activity against *E. coli* and *S aureus*. Thus, further investigations on the antibacterial activity of terpenoids and phytosterol separately or in combination are needed. Interestingly, the phytochemical investigation conducted on the chloroform leaf extract of *M. zavattarii* led to the isolation of two compounds namely, *β*-amyrin palmitate (compounds **1**) and lutein (compounds **2**) which are categorized under terpenoids, reported herein for the first time from this plant. Both *β*-amyrin palmitate and lutein showed stronger interaction with the 1G2A *protein of E. coli* than the chloramphenicol's interaction with the 1G2A protein target, but, paradoxically, chloramphenicol showed potent antibacterial activity against *E. coli*. Thus, the *in silico* molecular docking results need to be checked through experimental study including antibacterial activity of *β*-amyrin palmitate and lutein against *E. coli*. The drug-likeness property study indicated that both the isolated compounds violated two rules of Lipinski's rule of five with molecular weight (g/mol) >500 and LogP >4.15. To sum up, methanol and chloroform could be the preferred solvent for phytochemical extraction from the leaf of *M. zavattarii* for more bioactive phytochemical constituents screening and antibacterial activity test. Furthermore, further phytochemical investigation and biological activity should be conducted on the other parts of *M. zavattarii* as no other similar studies have been conducted on this endemic plant species of Ethiopia.

## Figures and Tables

**Table 1:** Preliminary phytochemical analysis of the leaf extract of *M. zavattarii.*

Phytochemical tested	Test performed	Extracts			
		*n*-hexane	CF	EtOAc	MeOH
Flavonoids	Alkaline (NaOH) reagent test Lead acetate test	−	+	−	++
Alkaloids	Mayer's and Wagner's reagents	−	−	+	−
Tannins	5% ferric chloride test	−	−	−	++
Terpenoids	Salkowski test	++	++	−	+
Saponins	Froth and foam test	−	+	−	++
Free anthraquinone	Borntrager's test	−	−	−	−
Phytosterols	Lieberman Bouchard test	+	+	+	+
Quinines	Ammonium hydroxide test	−	−	−	−
Phlobatannin	1% aqueous hydrochloric acid	−	+	−	−
Coumarin	Alcoholic NaOH test	−	+	−	+

*Note*. ++ = strong presence, + = moderate presence, − = absence, and CF = chloroform.

**Table 2:** Antibacterial activity of *M. zavattarii* crude extracts against human pathogenic bacteria.

Pathogens	Conc. (mg/mL)	Inhibition zone diameter in mm (mean ± SD) of extracts and standard			
		*n*-hexane	Chloroform	Methanol	Chloramphenicol
*E. coli*	50	7.67 ± 0.76	12.08 ± 0.38	7.00 ± 0.50	26.78 ± 0.63
	75	11.08 ± 0.80	14.00 ± 0.50	8.75 ± 0.25	
	125	14.75 ± 0.25	15.58 ± 0.63	11.00 ± 0.87	

*S. aureus*	50	10.33 ± 0.76	7.00 ± 0.50	7.25 ± 0.25	27.37 ± 1.84
	75	12.92 ± 0.63	7.17 ± 0.29	11.08 ± 0.38	
	125	14.08 ± 1.13	10.60 ± 1.50	16.42 ± 0.52	

*P. aeruginosa*	50	Nd	Nd	Nd	21.17 + 2.36
	75	Nd	Nd	Nd	
	125	Nd	Nd	Nd	

Nd = not detected.

**Table 3:** The ^1^H, ^13^C, and DEPT-135 NMR data (CDCl_3_) system of compound **1** and values in the literature for *β*-amyrin palmitate [21, 22].

Carbon no.	*δ* ^13^_C_	Compound **1**		*β*-amyrin palmitate	
		DEPT-135	*δ* ^1^_H_ (multiplicity and coupling)	*δ* ^13^_C_	*δ* ^1^_H_ (multiplicity and coupling
1	38.3	-CH_2_-	1.60–1.66 (1H, m)	38.2	1.60 (1H, m), 1.31 (1H, m)
			1.34−1.30 (1H, m)		
2	22.7	-CH_2_-	1.86–1.96 (1H, m)	22.7	1.85 (1H, m), 1.65 (1H, m)
			1.60–1.66 (1H, m)		
3	80.6	-CH-	4.50–4.54 (1H, m)	80.6	4.51 (1H, dd, *J* = 8.0, 7.5 Hz)
4	37.8	Q	—	37.7	
5	55.3	-CH-	0.89 (1H, m)	55.2	0.89 (1H, m)
6	18.3	-CH_2_-	1.58−1.54 (2H, m)	18.3	1.58 (1H, m), 1.54 (1H, m)
7	32.6	-CH_2_	1.27 (1H, m)	32.6	1.25 (1H, m), 1.15 (1H, m)
			1.15 (1H, m)		
8	39.8	Q	—	39.8	
9	47.2	-CH-	1.60–1.66 (1H, m)	47.2	1.60 (1H, m)
10	36.8	Q	—	36.8	
11	23.6	-CH_2_-	1.86–1.96 (H, m)	23.7	1.95 (1H, m), 1.63 (1H, m)
			1.60–1.66 (1H, m)		
12	121.7	-CH-	5.19 (1H, m)	121.6	5.20 (1H, t, *J* = 3)
13	145.2	Q	—	145.2	
14	41.7	Q	—	41.7	
15	26.9	-CH_2_-	1.15 (2H, m)	26.9	1.13 (2H)
16	26.1	-CH_2_-	1.27 (s, br)	26.1	1.25 (1H, m), 1.15 (1H, m)
			1.15 (1H, m)		
17	32.5	Q	—	32.4	
18	46.8	-CH-	1.86–1.96 (2H, m)	46.8	1.95 (1H, m)
19	47.6	-CH_2_-	1.86–1.96 (2H, m)	47.5	1.97 (1H, m), 1.63 (1H, m)
			1.60–1.66 (1H, m)		
20	31.1	Q	—	31.1	
21	34.9	-CH_2_-	(1.27, s, br)	34.7	1.45 (1H, m), 1.25 (1H, m)
			1.58−1.54 (1H, m)		
22	37.2	-CH_2_-	(1.27, s, br)	37.1	1.63 (1H, m), 1.25 (1H, m)
			1.60–1.66 (1H, m)		
23	28.4	-CH_3_	0.89 (3H, s)	28.4	0.89 (3H, s)
24	16.8	-CH_3_	0.85 (3H, s)	16.8	0.85 (3H, s)
25	15.6	-CH_3_	0.90 (3H, s)	15.5	0.89 (3H, s)
26	16.8	-CH_3_	0.98 (3H, s)	16.8	0.98 (3H, s)
27	25.9	-CH_3_	1.15 (3H, s)	25.9	1.14 (3H, s)
28	28.1	-CH_3_	0.85 (3H, s)	28.0	0.85 (3H, s)
29	33.3	-CH_3_	0.89 3H, s	33.3	0.89 (3H, s)
30	23.7	-CH_3_	0.89 (3H, s)	23.6	0.89 (3H, s)
1′	173.7	Q (C=O)	—	173.6	
2′	34.7	-CH_2_-	2.31 (2H, td, *J* = 7.8, 3)	34.9	2.30 (2H, dd, *J* = 7.6, 7.2)
3′	25.2	-CH_2_-	1.60–1.66 (2H,m)	25.2	1.60 (2H, m)
4′–13′	29.2′–29.7′	-CH_2_-	1.27 (24H, m overlapped)	29.2′–30.0′	1.26 (20H, m)
14′	31.9	-CH_2_-		31.9	1.26 (2H, m)
15′	23.5	-CH_2_-		23.5	1.28 (2H, m)
16′	14.1	-CH_3_	0.89 (3H, t)	14.1	0.88 (3H, m)

**Table 4:** ^1^H, ^13^C, and DEPT-135 NMR spectroscopic data (CDCl_3_) of compound **2** and values in the literature for lutein [27].

Carbon no.	Compound **2**			Lutein [27]	
	*δ* ^13^_C_	DEPT-135	*δ* ^1^_H_	*δ* ^13^_C_	*δ* ^1^_H_
1	37.1	Q	—	37.1	—
2	48.4	-CH_2_	1.49 (1H, t, *J* = 12.0)	48.4	1.48 (1H, t, *J* = 12)
			1.79 (H, d, *J* = 12.8)		1.77 (1H, dt, *J* = 11.9)
3	65.1	OCH	4.02 (1H, m)	65.1	4.00 (1H, m)
4	42.5	-CH_2_	2.28–2.44 (1H, m)	42.6	2.33–2.42 (1H, m)
			2.09 (1H, s)		2.04 (1H, dd, *J* = 10, 17
5	126.2	Q	—	126.2	—
6	138.0	Q	—	137.7	—
7	125.6	=CH	6.11–6.14 (1H, m)	125.6	6.12 (1H, s)
8	138.5	=CH	6.11–6.14 (1H, m)	138.5	6.12 (H, s)
9	135.7	Q	—	135.7	—
10	131.3	=CH	6.18 (1H, m)	131.3	6.15 (1H, m)
11	124.9	=CH	6.60–6.69 (1H, m)	124.9	6.57–6.66 (1H, m)
12	137.8	=CH	6.38 (d, *J* = 14.5 Hz, 1H)	137.7	6.36 (1H, d, *J* = 15)
13	136.5	Q	—	136.5	—
14	132.6	=CH	6.27 (1H, d, *J* = 8.3)	132.6	6.25 (1H, br, *J* = 9)
15	130.1	=CH	6.60–6.69 (1H, m)	130.1	6.57–6.66 (1H, m)
16	28.7	-CH_3_	1.09 (3H, s)	28.7	1.07 (3H, s)
17	30.3	-CH_3_	1.09 (3H, s)	30.3	1.07 (3H, s)
18	21.6	-CH_3_	1.75 (3H, s)	21.6	1.73 (3H, s)
19	13.1	-CH_3_	1.98 (3H, s)	12.8	1.97 (3H, s)
20	12.8	-CH_3_	1.98 (3H, s)	12.8	1.97 (3H, s)
1′	34.1	Q	—	34.0	—
2′	44.7	-CH_2_	1.86 (1H, dd, *J* = 13.1, 5.8)	44.6	1.84 (1H, dd, *J* = 6, 13)
			1.38 (1H, m)		1.37 (2H, dd, *J* = 7, 13)
3′	65.9	OCH	4.27 (1H, br. s)	65.9	4.25 (1H, m)
4′	124.5	=CH	5.56 (1H, s)	124.5	5.54 (1H, s)
5′	138.0	Q	—	137.8	—
6′	55.0	-CH	2.28–2.44 (1H, m)	55.0	2.33–2.42 (2H, m)
7′	128.7	=CH	5.44 (1H, dd, *J* = 15.4, 9.9)	128.7	5.43 (1H, dd, *J* = 10 15.5)
8′	138.5	=CH	6.18 (1H, m)	138.0	6.15 (1H, m)
9′	135.1	Q	—	135.1	—
10′	130.8	=CH	6.18 (1H, m)	130.8	6.15 (1H, m)
11′	124.8	=CH	6.60–6.69 (1H, m)	124.5	6.57–6.66 (1H, m)
12′	137.6	=CH	6.38 (d, *J* = 14.5 Hz, 1H)	137.6	6.36 (1H, d, *J* = 15)
13′	136.4	Q	—	136.4	—
14′	132.6	=CH	6.27 (1H, d, *J* = 8.3)	132.6	6.25 (1H, br, *J* = 9)
15′	130.1	=CH	6.60–6.69 (1H, m)	130.1	6.57–6.66 (1H, m)
16′	24.3	-CH_3_	0.86 (3H, s)	24.3	0.84(3H, s)
17′	29.5	-CH_3_	1.01 (3H, s)	29.5	1.00 (3H, s)
18′	22.9	-CH_3_	1.64 (3H, s)	22.8	1.63 (s)
19′	13.1	-CH_3_	1.92 (3H, br, s)	13.1	1.91 (3H, s)
20′	12.8	-CH_3_	1.98 (3H, s)	12.8	1.97 (3H, s)
3-OH	—	—	4.02 (s)	—	3.36 (s)
3′-OH	—	—	—	—	—

**Table 5:** Molecular docking results of isolated compounds **1** and **2** with peptide deformylase enzyme (PDF) (PDB ID: 1G2A) of *E.coli*.

Compounds	Binding energy (kcal/mol)	H-bonding distance (Å)	Interacting residues
**1**	−9.09	NI	Leu125, Ile44, Cys129, Glu88, His132, Arg97, Glu133, Glu87, Ile86, Gly89, Ile93, Cys90, Glu95, Leu91, Glu42, Glu41, and Ala40

**2**	−7.05	Cys129 (2.29), Glu133 (2.64), and Leu91 (2.44)	Glu88, Ile44, Arg97, Gly43, Glu42, His7, His132, Leu125, Gly89, Cys90, Glu41, and Pro94

Chloramphenicol	−6.87	Ile44 (2.89)	Gly89, Cys90, Glu42, Leu92, His132, Gly43, and Cys129
		Gly45 (2.97)	
		Gln50 (2.86), His136 (3.24), and Glu133 (3.30 and 3.32)	
		Arg97 (2.73)	

**Table 6:** Drug-likeness property of compounds **1** and **2** predicted by Swiss ADME software.

Compounds	Formula	Mol. wt. (g/mol)	NRB	NHA	NHD	TPSA (Å^2^)	LogP (cLogP)	Lipinski's rule of five violation	Bioavailability score
**1**	C_46_H_80_O_2_	665.13	16	2	—	26.30	12.42	2	0.17
**2**	C_40_H_56_O_2_	568.87	10	2	2	40.46	12.42	2	0.17
Chloramphenicol	C_11_H_12_Cl_2_N_2_O_5_	323.13	7	5	3	115.38	0.73	0	0.55

*Note*. NHD = number of hydrogen donor, NHA = number of hydrogen acceptor, NRB = number of rotatable bonds, and TPSA = total polar surface area and LogP (cLogP) = octanol-water partition coefficient.

**Table 7:** ADME property of isolated compounds **1** and **2** predicted by PreADMET software.

Compounds	Chemical formula	Log*K*_p_ (cm/s)	GI absorption	BBB permeability	Inhibitor interaction (swiss ADME/PreADMET)					
					P-gp substrate	CYP1A2 inhibitor	CYP2C19 inhibitor	CYP2C9 inhibitor	CYP2D6 inhibitor	CYP3A4 inhibitor
**1**	C_46_H_80_O_2_	1.75	Low	No	Yes	No	No	No	No	No
**2**	C_40_H_56_O_2_	1.75	Low	No	Yes	No	No	No	No	No
Chloramphenicol	C_11_H_12_Cl_2_N_2_O_5_	−7.46	High	No	No	No	No	No	No	No

*Note*. LogKp = skin permeation value, GI = gastro-intestinal, BBB = blood brain barrier, P-gp = P-glycoprotein, and CYP = cytochrome P.

**Table 8:** Toxicity property of isolated compounds **1** and **2** predicted by ProTox-II property software.

Compounds	Chemical formula	LD_50_ (mg/kg)	Toxicity class	Organ toxicity				
				Hepatotoxicity	Carcinogenicity	Immunotoxicity	Mutagenicity	Cytotoxicity
**1**	C_46_H_80_O_2_	339	4	No	No	Yes	No	No
**2**	C_40_H_56_O_2_	10	2	No	No	Yes	No	No
Chloramphenicol	C_11_H_12_Cl_2_N_2_O_5_	1500	4	No	No	No	Yes	No

## Data Availability

The data and materials that used to support the findings of this study are included within the manuscript.

## Supplementary Materials

Supplementary Materials Supplementary data showing the inhibition zone of antimicrobial activity have been attached.
