# Supplementary material for: Phytochemicals Analysis, In Vitro Antibacterial Activities of Extracts, and Molecular Docking Studies of the Isolated Compounds from Melhania zavattarii Cufod Leaves
Source: J Trop Med. 2023 Jun 1;2023:8820543. doi: 10.1155/2023/8820543 (PMC10250093; doi:10.1155/2023/8820543)
Supplement: Supplementary Materials — Supplementary data showing the inhibition zone of antimicrobial activity have been attached. [file 8820543.f1.docx]

**Supplementary Data**

**Phytochemicals analysis, in vitro antibacterial activities of extracts, and molecular docking studies of the isolated compounds from *Melhania zavattarii* Cufod leaves**


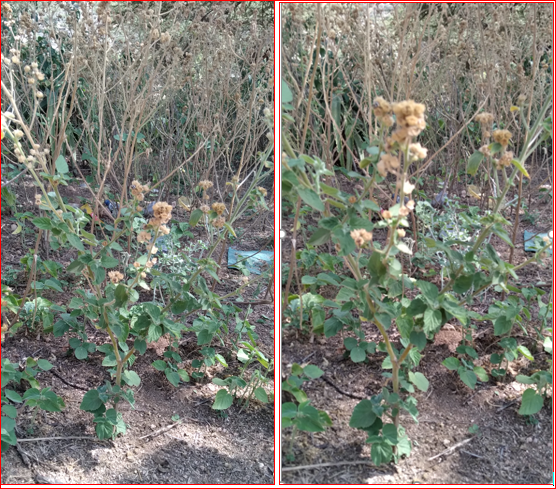


**Figure 1: Photo of Melhania zavattarii Cufod (Mukabira), December, 2021**
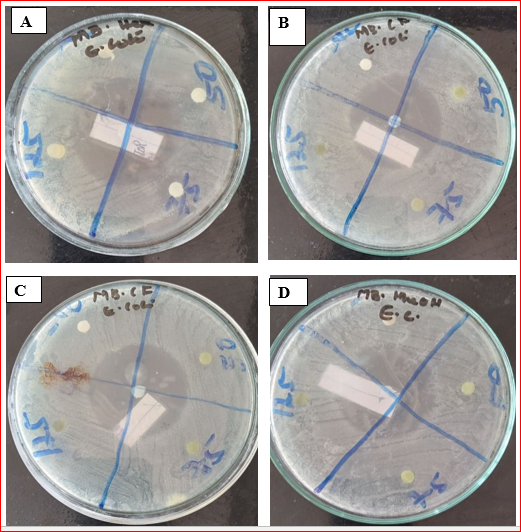


**B**


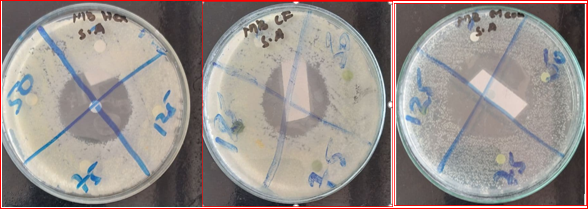


**B**

**B**

**A**

**Figure 2: Antibacterial activity of Leaf of Melhania zavattarii Cufod (Mukabira)**

**Figure 3: UV-visible absorption spectrum of Compound 1**

**Figure 4: IR spectrum of compound 1**

**Figure 5: ^1^HNMR spectrum of compound 1**

**Figure 6: ^13^C NMR spectrum of compound 1**

**Figure7: DEPT-135 spectrum of compound 1**

**Figure 8: UV-visible absorption spectrum of Compound 2**

**Figure 9: IR spectrum of compound 2**

**Figure 10: ^1^HNMR spectrum of compound 2**

**Figure 11: ^13^C NMR spectrum of compound 2**

An extra peak as impurity

s

**Figure 12: DEPT-135 spectrum of compound 2**
